# Preconcentration and Separation of Gold Nanoparticles from Environmental Waters Using Extraction Techniques Followed by Spectrometric Quantification

**DOI:** 10.3390/ijms231911465

**Published:** 2022-09-28

**Authors:** Ingrid Hagarová, Lucia Nemček, Martin Šebesta, Ondřej Zvěřina, Peter Kasak, Martin Urík

**Affiliations:** 1 Faculty of Natural Sciences, Institute of Laboratory Research on Geomaterials, Comenius University in Bratislava, Mlynská dolina, Ilkovičova 6, 845 15 Bratislava, Slovakia; 2 Department of Public Health, Faculty of Medicine, Masaryk University in Brno, Kamenice 5, 625 00 Brno, Czech Republic; 3 Center for Advanced Materials, Qatar University, Doha P.O. Box 2713, Qatar

**Keywords:** gold nanoparticles, separation, quantification, extraction techniques, spectrometric methods, environmental waters

## Abstract

The quantification of gold nanoparticles (AuNP) in environmental samples at ultratrace concentrations can be accurately performed by sophisticated and pricey analytical methods. This paper aims to challenge the analytical potential and advantages of cheaper and equally reliable alternatives that couple the well-established extraction procedures with common spectrometric methods. We discuss several combinations of techniques that are suitable for separation/preconcentration and quantification of AuNP in complex and challenging aqueous matrices, such as tap, river, lake, brook, mineral, and sea waters, as well as wastewaters. Cloud point extraction (CPE) has been successfully combined with electrothermal atomic absorption spectrometry (ETAAS), inductively coupled plasma mass spectrometry (ICP-MS), chemiluminescence (CL), and total reflection X-ray fluorescence spectrometry (TXRF). The major advantage of this approach is the ability to quantify AuNP of different sizes and coatings in a sample with a volume in the order of milliliters. Small volumes of sample (5 mL), dispersive solvent (50 µL), and extraction agent (70 µL) were reported also for surfactant-assisted dispersive liquid–liquid microextraction (SA-DLLME) coupled with electrothermal vaporization inductively coupled plasma mass spectrometry (ETV-ICP-MS). The limits of detection (LOD) achieved using different combinations of methods as well as enrichment factors (EF) varied greatly, being 0.004–200 ng L^−1^ and 8–250, respectively.

## 1. Introduction

Over the past few decades, nanoscience and nanotechnology have undergone a great expansion, which has led to the mass production and utilization of a wide variety of nanoscale materials. In general, a nanoscale object refers to any structure that ranges between 1 and 1000 nm in size [1]. The European Commission [2] defines nanomaterials as any manufactured or natural structures containing ≥50% particles having at least one dimension lying in the nanoscale range, i.e., below 100 nm. Moreover, a nanostructured material can also be considered any object with a larger size span, which exhibits distinct electronic or optical properties as a result of quantum confinement in at least one dimension [3].

Among the nanomaterials, metal-based nanoparticles have gained popularity because of their unique size-dependent physico-chemical properties [4]. Such structures include particles made of pure metals (e.g., Cu, Au, Ag, Fe) or their compounds (e.g., CuO, Au-CuO, Ag_2_S, FeS) [5,6,7]. Gold nanoparticles (AuNP) are amid the most frequently used ones. Because of their unique functional and tunable intrinsic electronic and optical properties [8,9] and easy and controllable preparation [10,11], they can be utilized in a wide range of applications [12], including future technologies such as nanosensors or biomedical drug delivery [13]. In the latter case, the AuNP can be conjugated with an antibody or with any functionalized moiety, including DNA, amino acids, therapeutic agents, or proteins, which extends their application to the biomedical field, e.g., as a biomolecular diagnostic tool [14,15,16]. Considering their extensive use and prospects for application in the medical and industrial sectors, we would like to reference some papers that address this topic [17,18,19,20,21] and to pull out some intriguing results and highlights.

The recent increase in use and disposal of AuNP seem to increase the risk of their intentional and unintentional release into the natural environment to such an extent that they are considered emerging pollutants [22,23,24]; their elevated concentrations can be expected in all environmental compartments [25]. Despite the increasing risk of exposure, the information on fate and toxicity of AuNP is still very limited [26,27], partly due to the lack of methods suitable for reliable AuNP quantification in complex matrices [28].

Since nanoparticles are considered unique analytes in terms of their chemical and physical features [29], the introduction of highly sophisticated methods is often necessary to achieve a satisfactory level of their characterization and quantification [30,31]. The basic structural characteristics of AuNP, such as size, shape, mono- or polydispersity, UV-Vis, along with some other spectral features and electrokinetic potential, provide important information relevant to the characterization of engineered nanomaterials. Thanks to remarkable advances in microscopic techniques, the surface morphology can be thoroughly studied by means of scanning electron microscopy (SEM), transmission electron microscopy (TEM), dark-field microscopy, and atomic force microscopy (AFM). A list of spectrometric techniques that are particularly applicable for analyzing a material’s surface chemistry and composition include UV-Vis spectrophotometry, Fourier-transform infrared spectroscopy (FTIR), X-ray fluorescence (XRF), X-ray photoelectron spectroscopy (XPS), and molecular fluorescence spectroscopy.

In the analysis of nanomaterials, quantitative information can be expressed via concentration metrics, such as (1) the mass concentration, (2) the surface area metric, and (3) the number concentration metric [22]. Due to relatively high limits of detection (LOD), many current quantification methods cannot be used unless preconcentration of the sample is carried out. Even when utilizing mass spectrometry-based methods that have emerged as a suitable alternative for reliable quantification of AuNP at the microgram-per-liter or even sub–microgram-per-liter levels (such as those expected or reported in aqueous environments) [32], co-existing matrix may pose a problem and so an effective separation technique needs to be involved.

Unfortunately, a reliable quantification of nanomaterials in any complex biological or environmental matrix is not an easy task. Low levels of monitored nanomaterials and complicated chemical composition of the hosting matrix may well be troubling for researchers trying to obtain reliable quantitative information on nanoparticles. To avoid this issue, a reliable quantification method should be combined with an appropriate separation technique. In order to provide more information on such procedures, this paper summarizes the recent studies in which well-established extraction techniques combined with commercially available spectrometric methods were utilized for the effective separation/preconcentration and reliable quantification of AuNP in natural aqueous media, wastewaters, soil extracts, and biological tissues. The benefits and drawbacks of particular extraction methods have been discussed in detail in our previous work [33].

With respect to the extraction phase, a sample preparation procedure can be performed using either liquid- or solid-phase extraction. The extraction techniques that utilize solid material as the extraction phase include solid-phase extraction (SPE), dispersive solid-phase extraction (dSPE), solid-phase microextraction (SPME), stir bar sorptive extraction (SBSE), magnetic solid-phase extraction (MSPE), thin-film microextraction (TFME), in-tube solid-phase microextraction (IT-SPME), and several others [33,34]. Conventional techniques based on the liquid extraction principle include traditional liquid–liquid extraction (LLE), also known as the solvent extraction. In order to reduce the solvent volume significantly, liquid-phase microextraction (LPME) has been developed from LLE through its miniaturization. In terms of separation mode, there are three main variants of LPME: single-drop microextraction (SD-LPME or SDME), dispersive liquid–liquid microextraction (DLLME), and membrane-mediated liquid-phase microextraction (e.g., hollow-fiber liquid-phase microextraction (HF-LPME) and solvent bar microextraction (SBME)) [33,35]. These methods have been modified and further developed in recent years [36,37].

The excessive use of organic solvents in analytical chemistry has prompted the development of cloud point extraction (CPE) technique, which employs nontoxic nonionic surfactants rather than organic solvents. This method has shown promising results even for the separation and preconcentration of metallic nanoparticles, including AuNP [6]. The optimistic prospects for extraction techniques coupled with spectrometric methods (e.g., UV-Vis spectrophotometry, electrothermal atomic absorption spectrometry, inductively coupled plasma mass spectrometry, chemiluminescence, total reflection X-ray fluorescence spectrometry, and surface plasmon resonance) for separation and preconcentration of metallic nanoparticles inspired us to review the recent achievements and the state-of-the-art in the field, with a focus on AuNP quantification in natural waters and wastewaters.

**Figure 1:**
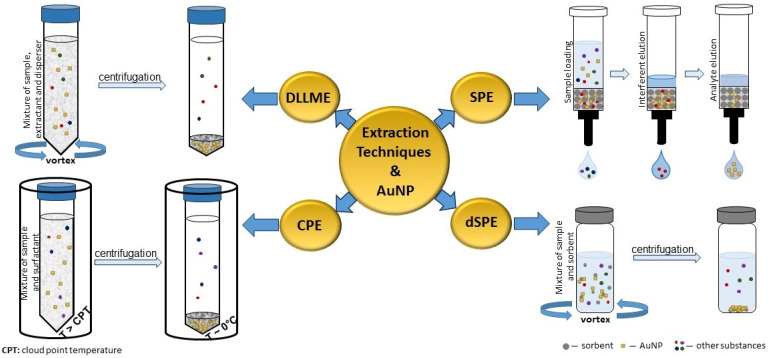
A schematic illustration of four extraction procedures recommended for separation and preconcentration of AuNP.

We conducted an overview of original research papers reporting on promising extraction techniques that allow the effective selective separation of AuNP in actual environmental matrices; we considered relevant articles published between 2009 and 2022. A scheme comparing four extraction procedures discussed in our paper is shown in the following figure (Figure 1).

## 2. Solvent Extraction Procedures

The metal ions and organic compounds have been traditionally separated from the aqueous matrices by means of liquid–liquid extraction (LLE). However, this technique utilizes large quantities of organic solvents, whose handling and disposal pose a significant environmental and health issue [38]. Since the technique is flexible enough to ensure isolation of a wide range of substances, the abovementioned drawback has had to be addressed and the LLE technique has been modified accordingly. The primary aim was to reduce the solvent volumes required for sample extraction and analysis to microliters. Liquid-phase microextraction (LPME) is a method that has undergone such modification and can be run under different extraction modes and conditions. A recently published overview by Câmara et al. [39] offers a detailed walk-through of the principles of several extraction formats of the LPME technique that have been utilized for separation and preconcentration of a diverse array of organic or inorganic analytes.

Later in this section, we will further discuss this unique technique that has been successfully applied to the selective separation of AuNP in analytically challenging matrices, such as chicken liver and natural waters of different composition.

### 2.1. Liquid-Phase Microextraction

In order to separate AuNP from liquid matrices, López-Lorente et al. [40] stabilized AuNP with a cationic surfactant hexadecyltrimethylammonium chloride (CTAC); such stabilized nanoparticles have been extracted into an ionic liquid phase by micro liquid–liquid extraction (IL-µLLE). The ionic liquids are organic salts that have been investigated as novel solvents for many applications. Ionic liquid-forming salts often display low-melting points and unique properties, including an extremely low vapor pressure, low viscosity, and high chemical, thermal, and electrochemical stability [41]. It is expected that this class of greener alternatives to organic solvents will be involved in the development of highly selective extraction procedures. From the ionic liquids tested by López-Lorente et al. [40], only those containing an imidazolium group were capable of extracting AuNP. An easy-to-synthesize-and-purify 1-butyl-3-methylimidazolium hexafluorophosphate (BMIM PF_6_) was eventually selected for the extraction of AuNP not only from river water, but also from chicken liver. Before adding 0.3 g of BMIM PF_6_ to 3 mL of river water specimen, samples were treated with 1.67 mM of CTAC and spiked with AuNP. The same amounts of surfactant and ionic liquid were used for the extraction of AuNP from chicken liver samples.

### 2.2. Dispersive Liquid–Liquid Microextraction

Since its introduction, dispersive liquid–liquid microextraction (DLLME) has undergone many modifications. There has been some development toward creating a new design of extraction devices and implementation of the new extractant dispersion strategies. There continues to be interest in new approaches to phase separation and in different types of extraction and dispersive solvents. In recent years, this separation technique has also been combined with some other extraction techniques to extend its application to more complicated matrices. These topics are discussed at length in a newly published paper by Sajid [42].

A ternary eutectic solvent-based DLLME technique allows for simultaneous extraction and preconcentration of analytes with organic solvents that are soluble in the dispersion medium and immiscible in water. This favors the formation of fine droplets of the extraction solvent. However, conventional DLLME requires high volumes of (usually hazardous) organic dispersants. Using large amounts of these chemicals also results in a decrease in magnitude of the partition coefficient of the analyte between the two phases of the extractant and an aqueous matrix.

The replacement of organic solvents with surfactants has allowed the development of more environmentally friendly DLLME modes, e.g., surfactant-assisted dispersive liquid–liquid microextraction (SA-DLLME). This mode has been successfully tested for separation of trace metals [43,44,45] and organic compounds [46,47,48]. It was also used for preconcentration and separation of AuNP by Liu et al. [28], who combined SA-DLLME with electrothermal vaporization inductively coupled plasma mass spectrometry (ETV-ICP-MS). In addition, the authors proposed the optimal experimental parameters for both efficient extraction (5 mL of water sample, 70 µL of extraction agent, and 50 µL of emulsifier) and reliable quantification of gold nanoparticles. Furthermore, there was no need for acid digestion or dilution of an extraction phase. For the purpose of this analysis, the complex aqueous matrices, including tap, lake, and river water samples, were examined in order to demonstrate the analytical potential of the method for real-world applications.

### 2.3. Cloud Point Extraction

The cloud point extraction (CPE) procedure is a green alternative methodology that circumvents the drawbacks of LLE. It replaces potentially toxic or carcinogenic organic solvents with neutrally charged nonionic or zwitterionic surfactants. The separation of an aqueous surfactant solution into two isotropic phases is possible because of changes in experimental conditions (mostly temperature, but also pressure, pH, and ionic strength). As a result of these changes, the solution becomes turbid due to an incomplete solubilization of the surfactant [5], forming micellar structures with the ability to entrap analyte molecules. Eventually, the surfactant phase of a small volume (surfactant-rich phase) is obtained, which not only allows for analyte separation, but also for the preconcentration of molecules [49]. In their literature overview, Mandal and Lahiri [50] provided a detailed insight into the CPE technique and its new modifications that have been used for the extraction of metal ions. We will address the CPE procedure and its utilization in the extraction of AuNP from natural water and wastewater in this part of the paper.

In their pioneering work on CPE, Liu et al. [51] evaluated the potential of this method for separation of nanoparticles (including AuNP) from aqueous matrices. Since then, CPE has been used with an array of detection methods for reliable quantification of selected nanoparticles, mostly in aqueous media.

CPE separation is often followed by an electrothermal atomic absorption spectrometry (ETAAS), a popular method that can be used for quantification. It requires only a few microliters of sample volume and no digestion of a surfactant-rich phase. The combination of these two procedures for the effective separation and reliable quantification of AuNP in waters containing both ionic gold and AuNP has been tested by Hartmann and Schuster [52]. By using sodium thiosulphate as a complexing agent, Au(III) species have been reduced to Au(I), which is the most stable oxidation state of gold. Moreover, thiosulphate forms a negatively charged and very stable gold complex [Au(S_2_O_3_)_2_]^3−^, which cannot be extracted by the surfactant; thus, only AuNP pass into the surfactant-rich phase. Besides evaluating the applicability of the method for nanoparticles of different origin, the effect of natural organic matter (NOM; simulated with commercially available humic acids) and inorganic colloids (simulated with amorphous TiO_2_ particles) on CPE efficiency has been studied in detail. Transmission electron microscopy (TEM) imaging confirmed that the particle size of AuNP was not affected by the CPE procedure.

An alternative approach has been proposed by Tsogas et al. [53], who combined the CPE procedure with chemiluminescence (CL) detection. The selective back-extraction method has been developed for sequential separation of AuNP, silver nanoparticles (AgNP), and magnetite nanoparticles (Fe_3_O_4_NP) from the micellar phase. This unique method allowed to distinguish between three types of engineered nanoparticles that were extracted from the same sample. This feature is highly valued, although the procedure itself is considered time-consuming.

Similarly, Bahadir et al. [54] have explored the feasibility of CPE combined with total reflection X-ray fluorescence spectrometry (TXRF) for the simultaneous quantification of AuNP and AgNP. This combination was tested on different types of spiked aqueous matrices, including tap water, seawater, and water collected from the river. Except for the water samples high in salts, it was not necessary to use a standard addition method for calibration purposes to achieve reliable results. The additional information on the effect of soil and organic matter content on AgNP and AuNP stability in soil extracts and water was also provided. Whereas the presence of humic acids resulted in AgNP dissociation, AuNP were less susceptible to degradation in water. The mobility of both nanoparticle types in soils is considered extremely low, therefore, less than 2% nanoparticles have been identified in soil aqueous extracts; the vast majority were found to be adsorbed onto the surfaces of soil constituents. Sample volumes were identical for all water specimens tested (10 mL).

El Hadri and Hackley [55] employed an optimized CPE procedure in an effort to extract AuNP from agricultural soil. The researchers investigated both the extraction efficiency and the size distribution of AuNP by means of a variety of spectrometric methods. For quantification of the total gold content, an inductively coupled plasma mass spectrometry (ICP-MS) was applied following acid digestion. The aggregation status and size distribution were evaluated using single-particle (sp)ICP-MS. After CPE, the spiked soil extracts were characterized by asymmetric flow field-flow fractionation (A4F). Based on the results and observations, it can be concluded that humic acid and polyvinylpyrrolidone coatings on the surface of nanoparticles contributed to a significant decrease in CPE recoveries in deionized water. This effect was particularly pronounced in experiments where a nonionic surfactant Triton X-114 was used with no additives (e.g., NaCl, citric acid, EDTA). However, in the agricultural soil extract, the extraction recovery for polyvinylpyrrolidone-coated AuNP was enhanced, most likely due to the presence of natural colloids that were co-extracted during CPE.

A new detection method that relies on an optical incoherent light scattering (OILS) of a nano-hybrid assembly formed by hydrogen bond interactions between AuNP and dithiothreitol-functionalized CdS quantum dots has been successfully tested for AuNP quantification by Mandyla et al. [56]. The experimental parameters affecting the extraction efficiency of AuNP were optimized and evaluated for AuNP of variable sizes and surface coatings. Water samples were collected from a local wastewater treatment plant in Greece, filtered, and stored at 4 °C until used for assay. A sample volume was as small as 9 mL, and the addition of sodium thiosulfate (200 µL of 0.25 M Na2S2O3) and saturated EDTA (500 µL) as masking agents permitted the accurate determination of AuNP in the presence of many foreign metal ions. The selectivity of the method towards gold ions and other nanoparticle species was also evaluated under different experimental conditions. Nanoparticles, such as citrate-capped AgNP and zinc oxide nanoparticles (ZnONP), have been used in interference experiments and their effect on the extraction recovery of AuNP has been investigated. While the interference of ZnONP at a concentration up to two-orders of magnitude higher than that of AuNP was less than 10%, citrate-capped AgNP caused issues even at concentrations that were the same than those of AuNP. The interferences of ionic metal species were completely alleviated after addition of thiosulphate and saturated solution of EDTA. Eventually, the proposed method was successfully applied in the analysis of AuNP in natural waters and wastewaters.

### 2.4. Suspended Aggregate Microextraction

An extraction procedure where ionic surfactants are utilized and more complicated supramolecular aggregates need to be prepared for entrapping the analytes of interest has been developed by Benedé et al. [57] and termed ‘in-situ suspended aggregate microextraction’ (iSAME). The extraction is carried out in a supramolecular aggregate phase, which is formed in situ in the sample solution through ion-association between two counter-ions. An aggregate phase is collected in the form of a thin film onto the surface of a plain filter paper by means of vacuum filtration. This approach was used for the extraction of AuNP from environmental samples by Choleva et al. [58]. The analytes were entrapped in a supramolecular aggregate phase composed of multilamellar vesicles, which was formed in situ in an aqueous sample solution through ion-association between a cationic surfactant (cetyltrimethylammonium bromide; CTAB) and a benzene sulfonic acid derivative (sulfosalicylic acid; SSA). After filtration, a thin film composed of supramolecular aggregates and AuNP was dried, peeled off, and dissolved in acidified methanol. The thus-obtained solution was subjected to ETAAS analysis. Recovery rates between 81 and 93%, a good precision, low LOD, and simplicity of the procedure indicate that this type of approach to the collection and extraction of AuNP from water has considerable merit.

## 3. Sorbent Extraction Procedures

A sample pretreatment by means of the solid-phase extraction (SPE) technique is achieved by partitioning of the analytes between the liquid mixture (sample) and the (ad)sorbent packed in a disc, syringe, well plate, or SPE pipette tip [59,60]. Dispersive solid-phase extraction (dSPE) offers an alternative approach to SPE. This straightforward sample preparation technique utilizes the direct dispersion of an insoluble solid sorbent in a sample solution under the aid of vortexing, sonication, or shaking. After a certain amount of time, the analyte-loaded sorbent is separated from the solution by centrifugation. Prior to quantitative instrumental analysis, the analytes are eluted from the media using a solvent that the analytes are soluble in [61].

The current trends in SPE techniques have been presented by Zhang et al. [62]. The authors conducted a thorough statistical analysis on all available studies published between 1978 and 2020 that revealed that both SPE and dSPE indeed are very popular and widely used separation techniques. In addition, the application of new extraction media and new separation technologies that have been proposed in the course of the last decade were also targeted in this review.

The utilization of SPE and dSPE in separation of AuNP from natural waters of different composition will be discussed in the following subsections.

### 3.1. Solid-Phase Extraction

Li et al. [63] evaluated the applicability of Amberlite IRN-78 anionic exchange resin for selective extraction of noble metal nanoparticles, including AgNP, AuNP, and palladium nanoparticles (PdNP), from natural waters. The mercaptosuccinic acid (MSA)-modified nanoparticles were reversibly loaded onto the resin via electrostatic interaction between the positively charged ammonium groups of Amberlite resin and carboxylic moieties of MSA. The authors suggested that the proposed method is highly beneficial because of the reversible character of the interaction that allows synthetic resin to be regenerated and reused for SPE.

Li and Leopold [64] suggested a two-step extraction procedure for separation of stabilized AuNP using C-18 reversed-phase silica gel (RP-C18) and an alkyl thiol 1-dodecanethiol (1-DDT) that allowed for quantitative adsorption and ligand-assisted extraction into the chloroform under ultrasonication, respectively. While the parameters and conditions in general (unoptimized) procedures for AuNP extraction varied greatly throughout the experiments in terms of the ‘enhancement’ employed, the parameters for conducting an optimized ligand-assisted extraction of AuNP from RP-C18 material were as follows: concentration of 1-DDT was 10 mM, ultrasonication time was 3 h, and volume ratio of source to extractant was 1:1. The method was successfully tested for AuNP selective extraction from aqueous media containing gold ions, of which less than 0.35% have been extracted into chloroform, thus demonstrating good prospects of this method for actual use.

The hydrophilic polymer monolithic capillary (poly(acrylamide-vinylpyridine-methylene bis-(acrylamide)), poly(AA-VP-Bis)) prepared by Zhang et al. [65] has been used for separation and preconcentration of carboxyl group-containing AuNP from environmental water samples, followed by ICP-MS quantification. The benefits related to utilization of a monolithic polymer column were stressed in this paper as well as the advantages of capillary microextraction (CME) over the other microextraction techniques. The quantitative elution was attained using cysteine and cysteamine. Eventually, cysteamine was preferred as the eluent due to its better solubility and due to concerns surrounding AuNP aggregation in the presence of cysteine under certain conditions. Furthermore, the monolithic capillary was effectively regenerated by passing through 0.1 mL of 4% (*m/v*) cysteamine at a flow rate of 0.05 mL min^−1^. After regeneration, the DIY polymer monolithic capillary could be reused 20 times without any obvious decrease in extraction efficiency.

### 3.2. Dispersive Solid-Phase Extraction

The dSPE method has attracted considerable attention mainly because of its relatively simple design, short extraction time, and very low volume of liquid agents required. Two other factors that contribute to the growing popularity of the technique are the high efficiency of the developed procedures and their broad applicability. Moreover, this method embraces the use of diverse materials. A paper by Ścigalski a Kosobucki [66] gives an overview of materials currently being used in dSPE. The following paragraphs detail the application of two interesting sorbents for separation and preconcentration of AuNP in dSPE procedures.

A simultaneous extraction of both AuNP and gold ions from the aqueous media using dissolvable layered double hydroxides (LDH) has been reported by Choleva and Giokas [67]. The layered structure was composed of positively charged mixed magnesium–aluminum hydroxides, and intercalated charge-compensating anions and water molecules. While AuNP have been extracted via electrostatic interactions with LDH, the gold ions were preferentially extracted via ion-exchange mechanism. Subsequent ultracentrifugation allowed for selective quantification of AuNP in the samples. The efficiency of the LDH in extracting AuNP from water was optimized by investigating the concentration and ratio of the precursor metal ions (Mg^2+^ and Al^3+^), concentration of KOH, extraction time and temperature, and parameters related to the collection and elution of AuNP (i.e., centrifugation and elution steps). The optimization experiments were performed in 10 mL aqueous solutions spiked with 15.8 nM citrate-capped AuNP (4 nm).

Sulfonated nanocellulose (s-NC) has been used as an ecofriendly sorbent for the extraction of AuNP in combination with surface plasmon resonance (SPR) measurements in a study by Jesús Dueñas-Mas et al. [68]. The affinity of sulfur atoms towards metals appeared to be an important factor in the removal of metals from the aqueous phase. The stabilization of AuNP by cationic surfactant (cetyltrimethylammonium chloride; CTAC) considerably improved the extraction yields. Special attention was given to some other metal-based nanoparticles, such as titanium dioxide nanoparticles (TiO_2_NP) and plasmonic AgNP. Whereas TiO_2_NP were adsorbed onto s-NC and no elution was observed under procedural conditions, citrate-coated AgNP were retained by the sorbent and eluted. Since citrate-coated AgNP and AuNP have SPR absorption bands in different wavelength regions, 400 and 527 nm, respectively, AuNP could have been easily identified.

### 3.3. Magnetic Solid-Phase Extraction

There is a variety of natural and synthetic materials that have been used as absorbing agents. Recently, there has been much interest in integrating new advanced materials such as nanomaterials as potential adsorbents into dSPE procedures. However, ultracentrifugation and filtration of samples containing nanoparticles is laborious and has been challenged by relatively low sample yields, which makes the entire extraction process much slower. Therefore, a new method has been developed. Magnetic solid-phase extraction (MSPE) offers several advantages over conventional SPE, including convenient separation of magnetizable sorbent through the application of an external magnetic field [69].

The MSPE technique can be utilized for separation of organic molecules and ultratrace elements from different types of matrices. A recently published review by Ricardo et al. [70] focuses on the use of MSPE in speciation analysis of trace elements, especially Cr, Hg, As, and Se; these four elements have been identified as the most frequently analyzed, according to the literature. As a minor point, the authors mention the selective separation of AgNP in the presence of Ag^+^ ions using an optimized MSPE procedure. This paper suggests that a trend has emerged towards the use of this separation technique in the selective separation and preconcentration of metal nanoparticles. Some examples of utilization of MSPE in the selective separation and preconcentration of AuNP from real aqueous matrices will be presented in the following paragraphs.

The nanoparticles were extracted from an aqueous mixture of gold ions and AuNP with the assistance of Al^3+^-immobilized Fe_3_O_4_@SiO_2_@iminodiacetic acid (Fe_3_O_4_@SiO_2_@IDA–Al^3+^) composite sorbent; the recommended amount of sorbent was 20 mg for a 5 mL sample volume [71]. Both gold species were simultaneously immobilized by the composite, however, the following sequential elution of gold ions and AuNP with Na_2_S_2_O_3_ and NH_3_.H_2_O, respectively, allowed for their successful separation. There was a variety of AuNP sizes (14–140 nm) and shapes tested, and their dimensions and morphology remained unchanged after procedure. Furthermore, the authors highlighted that the coatings on the surface of AuNP (including citrate, 11-mercaptoundecanoic acid (MUA), polyvinylpyrrolidone (PVP), and cetyltrimethylammonium bromide (CTAB)) did not affect the separation and preconcentration efficiencies. The integration of all the aforementioned aspects of the method allowed for direct introduction of elution solutions to the mass analyzer (ICP-MS) and no digestion was required.

García-Figueroa et al. [72] described a method for the extraction of AuNP and gold ions using naked Fe_3_O_4_NP. A 0.5-mL aliquot of a 1 mg mL^−1^ sorbent suspension with 0.5 mL of ascorbic acid (1 M solution) was added to 5 mL of the sample solution. While the use of ascorbic acid allowed for quantitative recoveries of both species, a pre-reduction step using thiosulfate was successful enough for selective extraction of AuNP. The excellent performance of this method was compounded by the fact that there were no significant differences in the extraction of AuNP of different sizes, morphologies, and surface coatings. Furthermore, the direct injection of the analyte-loaded Fe_3_O_4_NP sorbent into ETAAS circumvents the laborious process of digestion and/or dilution prior analysis.

The attributes of different types of interactions that are responsible for the ability to selectively separate AuNP from mixtures containing various interfering ions, nanoparticles, and some other compounds, and the examples of the tolerance limits reported for several coexisting components of interest, will be presented at the beginning of the following section. The possible interferents that may, or are likely to, be present in environmental samples, and are expected to pose a barrier that inherently limits the selective separation of AuNP, will also be discussed.

## 4. Summary

The stabilization of AuNP is very important for the development of a reliable extraction procedure. Coating the surface with either negatively or positively charged small molecules results in the electrostatic stabilization of nanoparticles. Citrate, whose carboxyl groups play a significant role in the electrostatic stabilization of AuNP, is a good example of a small molecule. Larger polymer molecules, such as polyvinylpyrrolidone (PVP) or polyvinylalcohol (PVA), which are also frequently used for AuNP stabilization, can sometimes pose a problem due to steric repulsion forces between adjacent particles. When using cysteine, which contains both free carboxyl and amine functional groups, it should be considered that AuNP stabilized in this way may become positively charged under certain conditions. The positive effect of cationic surfactants (CTAC, CTAB) has been demonstrated during stabilization of AuNP in dSPE procedure with s-NC as a sorbent [68], and also in the µLLE procedure during which AuNP were extracted into an ionic liquid BMIM PF_6_ (1-butyl-3-methylimidazolium hexafluorophosphate) [40].

In CPE procedures, hydrophobic compounds are expected to be extracted into the surfactant-rich phase more easily than hydrophilic compounds. However, this was not the case with PVP and humic acid (HA) involved in AuNP stabilization [55]. Although PVP-AuNP and HA-AuNP contain a long hydrocarbon chain and in HA-AuNP also aromatic groups are present, which makes them more hydrophobic than citrate-capped AuNP, the latter were extracted more efficiently.

Positive interactions between AuNP and the surface groups of the sorbent as well as the high affinity of AuNP towards functional groups of the liquid extraction agent are another important indicator that is responsible for the effective separation of AuNP. Utilization of Fe_3_O_4_@SiO_2_@IDA–Al^3+^ sorbent has been shown to be associated with several different interactions responsible for the quantitative adsorption of AuNP; these interactions largely depend on the technique for nanoparticle stabilization and on the pH of the analyzed solution [71]. It has been demonstrated that simple electrostatic interactions between positively charged ‘naked Fe_3_O_4_NP’ and negatively charged AuNP did not favor quantitative extraction of AuNP [72]. Nonetheless, the addition of ascorbic acid improved the extraction yields significantly (>90%). Utilization of LDH (layered double hydroxides) as adsorbents revealed two types of interactions that were responsible for selective separation of AuNP in the presence of gold ions: electrostatic interactions between AuNP and LDH, and ion-exchange between gold ions and LDH [67]. In order to apply nanocellulose (NC) as an effective sorbent for AuNP, its surface modification was necessary [68]. The interaction based on positive affinity of sulfur atoms to gold played a decisive role in the modification of nanocellulose surface with sulfonate groups (s-NC).

The possible interferences that could be encountered in the analysis due to the presence of interfering substances in the matrix are outlined in the following text.

Based on the literature, it can be concluded that inorganic ions (either cations or anions) commonly found in water did not interfere with the selective separation of AuNP. However, this does not apply when using LDH [67]. LDH represent a positively charged sorbent, which can potentially be affected by an ionic strength of the analyzed solution. An increase in ionic strength is associated with an excess of anions in the solution and their adsorption on the positively charged surface of LDH, which results in a change in LDH surface charge. The overall reduction in extraction yields at high concentrations of inorganic ions can be explained either by counterbalancing the negative surface charge of AuNP, which may facilitate their aggregation and reduce their electrostatic interaction with the positively charged LDH surface, or by affecting the structure and morphology of the LDH as well as the selectivity of anion exchange [67].

The ionic forms of gold [28,53,67], some other metal nanoparticles (e.g., AgNP, TiO_2_NP, ZnONP, PdNP) [28,53,56,68], and natural organic matter (NOM, mostly simulated by adding HA) [28,71,72] have generally received major attention in the interference studies. The co-extracted ionic forms of gold (Au(III)) have become a serious issue both in SA-DLLME [28] and CPE procedures [52,54]. In order to separate Au(III) from AuNP, all authors prioritized sodium thiosulfate (Na_2_S_2_O_3_), which was added to reduce Au(III) to Au(I). The subsequently formed complex [Au(S_2_O_3_)_2_]^3−^ could not partition into the extraction phase, which resulted in the selective separation of AuNP from ionic forms of gold.

The presence of AgNP as a model interferent did not produce any false-positive or false-negative results in terms of the ability to detect the analyte in SA-DLLME [28], not even at 100-fold excess of the interferent in relation to the concentration of the analyzed compound. TiO_2_NP, which were used as model interferents in a study focused on possible interferences in CPE procedure [53], were shown to have negligible impact on the AuNP extraction up to a TiO_2_NP concentration of 50 mg mL^−1^. The effect of NOM modeled by commercially available HA on the extraction yields in CPE procedure was not significant at HA concentrations below 10 mg mL^−1^ [53]; in SA-DLLME, the extraction recoveries of AuNP were not affected by the addition of HA when their concentration did not exceed 30 mg mL^−1^ [28]. This value also represents a tolerance limit for HA [28,71]. The tolerance limits for ions frequently present in natural waters differed between publications. In regard to K^+^, Na^+^, Ca^2+^, Mg^2+^, NO^3−^, SO_4_^2−^, and Cl^−^, some interference studies have reported very high concentration values in the order of thousands of mg mL^−1^ [28,71,72]. The tolerance limits for other possible interferents were as follows: <1 mg mL^−1^ for Fe^3+^, Cu^2+^, Cd^2+^, and Pb^2+^ [71]; < 25 mg mL^−1^ for Zn^2+^, Cu^2+^, Ni^2+^, and Al^3+^ [58]; and < 10 mg mL^−1^ for inorganic ions such as PO_4_^3−^ and F^−^ [58]. Even at concentration levels that were far in excess of those of AuNP, these ions did not influence the proposed extraction procedures.

Based on the results of procedures discussed in this paper, it is apparent that both liquid- and solid-phase extractions can be used for the effective separation and preconcentration of AuNP from water samples of various complexity (such as tap, river, lake, brook, mineral, and seawater, as well as influent and effluent wastewater). The effectiveness of procedures was tested by analyzing an array of water samples spiked with known amounts of AuNP, often at two to three different concentration levels. In one particular case, the accuracy and precision of the method were evaluated by analyzing river water samples spiked at six concentration levels [40]. The concentration of AuNP in all analyzed samples was under the LOD of the analytical methods, except for untreated urban water samples where the concentrations of AuNP were found to be in the range of 10.8–24.4 ng L^−1^ [52]. Besides water samples, the liver homogenate [40] and agricultural soil extracts [55] were also analyzed to demonstrate the analytical potential of proposed extraction procedures to selectively separate AuNP from complicated matrices.

In papers with a special focus on the size and shape of AuNP during the extraction process, a solid-state analysis revealed that these physical properties remained unaffected [52,71,72]. Some AuNP were coated in order to demonstrate the method’s applicability over a broad range of coatings [56,58].

The extraction recovery rates reported in the reviewed studies were acceptable (Table 1), except for salty water samples analyzed by Bahadir et al. [54]. In this case, recoveries ranged from 40 to 50%, and the standard addition method had to be applied for calibration to get sufficiently accurate and precise results. The precision of the developed procedures, expressed by the relative standard deviation (RSD), was found to be acceptable in all reviewed papers (Table 1). Based on this parameter, it can be concluded that methods employed in preconcentration and separation of AuNP from selected matrices are reproducible.

The accuracy of novel analytical methods should be verified or validated using certified reference materials (CRM). However, there is a lack of commercially available CRM for environmental matrices containing known concentrations of AuNP; therefore, the accuracy of measurements was tested by the analysis of environmental samples spiked with known amounts of AuNP. In all reviewed papers, the extraction yields were used for validation of the methodology and for accuracy checks. Some information on the reference materials (RM) of AuNP is available on the National Institute of Standards and Technology (NIST) website. The reference materials termed as RM 8011, 8012, and 8013 consist of 5 mL of citrate-stabilized AuNP in an aqueous suspension sterilized by gamma irradiation, which are 10, 30, and 60 nm in size, respectively. However, these RM were developed especially for evaluating and qualifying the instrument performance and methodology related to the dimensional characterization of nanoparticles often used in clinical biomedical research [73]. Unfortunately, recent information on their availability reveals that RM 8012 and RM 8013 are out of stock and their production was discontinued. If production is resumed at some point, utilizing these materials in extraction procedures for spiking of samples will be considered again. Nonetheless, they cannot replace CRM and make no endeavor to do so.

The limits of detection (LOD) and enrichment factors (EF) varied greatly between publications (Table 1). Although the detection power of a quantification method is one of the decisive parameters in terms of sensitivity, with the right choice of methods it is possible to obtain comparable results. For instance, LOD achieved using a combination of CME and ICP-MS [65] and a combination of dSPE and ETAAS [67] can be considered comparable, despite the fact that ICP-MS has a better sensitivity than ETAAS. The EF were in the range of 8–250 (Table 1). In some extraction procedures, this parameter could be optimized by increasing the initial sample volume and/or by decreasing the eluting agent volume.

It has become obvious that the role of surfactants in extraction processes is crucial; they are an inherent part of all liquid-phase extractions described in the text. In a nonionic form, these reagents are mainly used in CPE procedures as extractants [52,53,54,56]. In these types of extractions, AuNP are being trapped in micellar structures formed with surfactant monomers. The nonionic surfactants found their application also in SA-DLLME procedures, as dispersants [28]. There is another group of surfactants in which cation is a surface-active component. Cationic surfactants can be utilized in IL-µLLE procedures as auxiliary agents for AuNP stabilization [40] or in iSAME procedures, in which AuNP are entrapped in a supramolecular aggregate phase [58]. In solid-phase extractions, surfactants serve only as auxiliary agents, mostly for AuNP stabilization. Cationic surfactants were also adopted in the dispersive arrangement of SPE, specifically, cetyltrimethylammonium chloride in standard dSPE [68] and cetyltrimethylammonium bromide in MSPE [71]. The utilization of non-toxic reagents, such as surfactants, in the newly developed extraction procedures represents a green analytical approach that eliminates the use of hazardous organic solvents. Nowadays, the current trend towards green chemistry is apparent in all chemical disciplines, extraction techniques included.

## 5. Conclusions

Due to the extensive use of AuNP in biomedical and industrial applications, they have become pollutants and their concentrations are expected to grow in all environmental compartments. In an effort to understand their fate and behavior in bio-geo-chemical cycles, it is important to know their concentration levels in environmental media. There are several analytical techniques and methods that can be used to detect and quantify AuNP. Some of them are quite sophisticated and very expensive. This paper offers an overview of cheaper alternatives to address the problem of gathering reliable quantitative information on ultratrace concentrations of AuNP in actual water samples of various complexity. The quantification of AuNP in tap, river, lake, brook, mineral, and seawater, as well as in influent and effluent wastewater samples, has been well documented. Using well-established extraction techniques coupled with common spectrometric methods, the reliable results have been reported by all authors. The optimization of experimental conditions resulted in high enrichment factors and recovery rates, low limits of detection, and a high level of precision.

The articles included in this review emphasize that the proposed extraction procedures allow for the selective separation of AuNP in the presence of gold precursor metallic ions (Au(I), Au(III)) and/or in the presence of other metal-based nanoparticles. In order to simulate a complicated co-existing matrix, a large volume of natural organic matter was added to a model solution to assess the separation efficiency. Since the chemistry of AuNP transformation in these matrices is not fully understood yet, and conversion of the analyte (which involves changes in size, shape, coating, etc.) may take place, a thorough systematic research on the involvement of potential interfering ions should be conducted. In spite of a range of manufacturers and suppliers of certified reference materials (CRM) which are the primary tools for analytical method validation, the reference materials of environmental matrices certified for their AuNP content are still lacking. In this regard, collaboration between the analytical scientists and other professionals could be an effective way of developing reliable CRM and applying them in new and interesting areas. Although there is a great shortage of CRM, with the right combination of extraction procedures and spectrometric methods it is possible to separate, preconcentrate, and quantify AuNP in real aqueous matrices. The vast analytical potential and applicability of these techniques were proven by the analysis of liver homogenate and agricultural soil extracts.

## Figures and Tables

**Table 1:** Performance data obtained in the experiments on separation/preconcentration and quantification of AuNP in water samples by extraction and spectrometric methods.

Extraction Technique	Water Samples	Detection Method	EF	LOD (ng L^−1^)	RSD (%)	Recovery (%)	Reference
IL-µLLE	River water	UV-Vis	NR	0.335	18.0	79–103	[40]
SA-DLLME	Tap, lake, river water	ETV-ICP-MS	152	2.20	9.3	90–102	[28]
CPE	River water, influent and effluent wastewater	ETAAS	80	5.00	9.5	91–103	[52]
CPE	Lake, river water, influent wastewater	CL	NR	0.217	2.3–12.4	79–114	[53]
CPE	Tap, river, sea, mineral water	TXRF	NR	200	9.6–16.0	90–102	[54]
CPE	River, lake water, effluent wastewater	OILS	NR	0.114	9.3	79–110	[56]
iSAME	Tap, river water, effluent wastewater	ETAAS	8	0.015	5.4–12.0	81–93	[58]
SPE	River, lake, brook water	ETAAS	132	NR	NR	62–69	[63]
SPE	Tap, river, lake, brook water, effluent wastewater	UV-Vis	250	NR	NR	68–99	[64]
CME	Tap, river, lake water	ICP-MS	10	0.005	5.6	77–103	[65]
dSPE	River, lake water, effluent wastewater	ETAAS	NR	0.004	7.8–8.9	71–92	[67]
MSPE	Sea water, lake, river, sewage water	ICP-MS	50	0.31	4.9	73–100	[71]
MSPE	Sea water, surface, ground water, artificial wastewater	ETAAS	199	19.5	5.3	85–98	[72]

NR: not reported; EF: enrichment factor; LOD: limit of detection; RSD: relative standard deviation.

## Data Availability

Not applicable.

## Abbreviations

1-DDT	1-dodecanethiol
A4F	asymmetric flow field-flow fractionation
AFM	atomic force microscopy
AgNP	silver nanoparticles
AuNP	gold nanoparticles
BMIM PF_6_	1-butyl-3-methylimidazolium hexafluorophosphate
CL	chemiluminescence
CME	capillary microextraction
CPE	cloud point extraction
CRM	certified reference material
CTAB	cetyltrimethylammonium bromide
CTAC	cetyltrimethylammonium chloride
dSPE	dispersive solid-phase extraction
DLLME	dispersive liquid-liquid microextraction
EDTA	ethylenediaminetetraacetic acid
EF	enrichment factor
ETAAS	electrothermal atomic absorption spectrometry
ETV-ICP-MS	electrothermal vaporization inductively coupled plasma mass spectrometry
Fe_3_O_4_@SiO_2_@IDA–Al^3+^	Al^3+^-immobilized on core-shell structure of Fe_3_O_4_ and SiO_2_ microspheres functionalized with iminodiacetic acid
Fe_3_O_4_NP	iron oxide nanoparticles
FIA	flow injection analysis
FTIR	Fourier-transform infrared spectroscopy
HA	humic acid
HF-LPME	hollow-fiber liquid-phase microextraction
ICP-MS	inductively coupled plasma mass spectrometry
IL-µLLE	micro liquid-liquid extraction in an ionic liquid
iSAME	in situ suspended aggregate microextraction
IT-SPME	in-tube solid-phase microextraction
LDH	layered double hydroxides
LLE	liquid–liquid extraction
LOD	limit of detection
LPME	liquid-phase microextraction
MSA	mercaptosuccinic acid
MSPE	magnetic solid-phase extraction
MUA	11-mercaptoundecanoic acid
NOM	natural organic matter
OILS	optical incoherent light scattering
PdNP	palladium nanoparticles
PVA	polyvinylalcohol
PVP	polyvinylpyrrolidone
poly(AA-VP-Bis)	poly(acrylamide-vinylpyridine-methylene bis-(acrylamide)
PR-C18	C-18 reversed-phase silica gel
RSD	relative standard deviation
SA-DLLME	surfactant-assisted dispersive liquid-liquid microextraction
SBME	solvent bar microextraction
SBSE	stir bar sorptive extraction
SDME	single drop microextraction
SEM	scanning electron microscopy
s-NC	sulfonated nanocellulose
SPE	solid-phase extraction
(sp)ICP-MS	single particle inductively coupled plasma mass spectrometry
SPME	solid-phase microextraction
SPR	surface plasmon resonance
SSA	sulfosalicylic acid
TEM	transmission electron microscopy
TFME	thin-film microextraction
TiO_2_NP	titanium dioxide nanoparticles
TS	thiosulphate
TXRF	total reflection X-ray fluorescence spectrometry
UV-Vis	UV/Vis spectrophotometry
XPS	X-ray photoelectron spectroscopy
XRF	X-ray fluorescence
ZnONP	zinc oxide nanoparticles
